# Antifungal Activity of a Medical-Grade Honey Formulation against *Candida auris*

**DOI:** 10.3390/jof7010050

**Published:** 2021-01-13

**Authors:** Theun de Groot, Tom Janssen, Dirk Faro, Niels A. J. Cremers, Anuradha Chowdhary, Jacques F. Meis

**Affiliations:** 1 Department of Medical Microbiology and Infectious Diseases, Canisius Wilhelmina Hospital (CWZ), 6532 SZ Nijmegen, The Netherlands; tom.janssen2511@hotmail.com (T.J.); dirk.faro@gmail.com (D.F.); jacques.meis@gmail.com (J.F.M.); 2 Triticum Exploitatie BV, Sleperweg 44, 6222 NK Maastricht, The Netherlands; niels@mesitran.com; 3 Department of Medical Mycology, Vallabhbhai Patel Chest Institute, University of Delhi, Delhi 110007, India; dranuradha@hotmail.com; 4 Centre of Expertise in Mycology Radboudumc/CWZ, 6532 SZ Nijmegen, The Netherlands; 5 Bioprocess Engineering and Biotechnology Graduate Program, Federal University of Paraná, Curitiba 80060-000, Brazil

**Keywords:** *Candida auris*, candidiasis, infection, genotyping, antifungal resistance, medical-grade honey, alternative therapy

## Abstract

*Candida auris* is a pathogenic yeast causing outbreaks in intensive care units with high mortality rates. The treatment of *C. auris* colonization is challenging due to high resistance rates. A potential alternative antifungal treatment is medical-grade honey. In this study the susceptibility of *C. auris* and other *Candida* species to the medical-grade honey-based formulation L-Mesitran^®^ Soft was investigated. The medical-grade honey formulation reduced the growth of *C. auris* and other *Candida* species in a dose-dependent manner. This inhibition was not only due to the honey component, as treatment with an identical concentration of this component only was less effective and even stimulated the growth of *C. albicans* and *C. glabrata*, supporting the interpretation that supplements in the medical-grade honey formulation enhanced the antimicrobial activity. Increasing the concentration of the honey component to 40%, as is also present in an undiluted medical-grade honey formulation, lead to a 1- to 4-log inhibition of all *Candida* species. Unprocessed local honey reduced the growth of nearly all *Candida* species more strongly than medical-grade honey. *C. auris*’ susceptibility to the medical-grade honey formulation did not depend on geographic origin or resistance profile, although the multiresistant isolates tended to be more susceptible. Altogether, medical-grade honey formulation has a strong antifungal activity against *C. auris* and other *Candida* species. Future studies should demonstrate whether the treatment of open wounds or skin colonized with *C. auris* is feasible and effective in the clinical setting.

## 1. Introduction

*Candida* species are the most common cause of fungal infections in humans. While various *Candida* species colonize mucosal surfaces and skin, this colonization does not lead to disease in healthy individuals [1]. Injury to the mucosal or skin barrier or a compromised immune system can lead to the invasion of these yeast and cause systemic infections. The majority of candidiasis cases worldwide is caused by a small number of species, including *C. albicans*, *C. glabrata*, *C. tropicalis*, *C. parapsilosis*, and *C. krusei* [2]. Over the last few years, *C. auris*, a recently emerged species, is increasingly contributing to invasive *Candida* infections. Moreover, this species has been highly contagious, resulting from the spread from just a few to many locations throughout the world within a decade [3]. *C. auris* caused nosocomial outbreaks in six continents with mortality rates up to 60% at intensive care units [4,5]. Although such patients are already severely ill, it demonstrates the high pathogenicity of this yeast. Whole-genome sequencing has demonstrated that *C. auris* can genotypically be divided into five different clades, from South Asia (I), East Asia (II), Africa (III), South America (IV), and Iran (V) [6,7]. While clades I–IV have all spread to different continents, only one isolate has been identified from the fifth clade until now [6]. 

The majority of *C. auris* isolates are resistant to fluconazole, the standard antifungal agent [3,8]. This is mainly due to the Y132F and K143R mutations in the *ERG11* gene, which encodes for lanosterol 14α-demethylase [5,8]. This enzyme enables the production of ergosterol, the target of azole agents. Both mutations make this enzyme less sensitive to for azole inhibition [5,8]. Additionally, some isolates also developed resistances to amphotericin B and, to a lesser extent, even to echinocandins [5,8]. While the molecular cause for amphotericin B resistance is still elusive, echinocandin resistance has been repeatedly associated with the S639F mutation in the *FKS1* gene, which encodes for an enzyme involved in the production of the polymer 1,3-beta-D-glucan, an important component of the fungal cell wall that is targeted with echinocandins [8,9]. The S639F mutation blocks the echinocandin-induced inhibition of this enzyme. Treatment options are limited by such multiresistant isolates [3,8]. 

Due to the limited treatment options of multiresistant isolates, it is essential to expand treatment options. A promising candidate for the treatment of yeasts in wounds and skin colonization is honey. Medical-grade honey follows strict criteria to guarantee its safety, quality, and efficacy, and was also found to be effective in inhibiting different *Candida* species, such as *C. albicans* and *C. tropicalis* [10,11,12,13]. Medical-grade honey consists of more than 200 different components influenced by its botanical origin, geographical location and secretions from the bee [14]. The antimicrobial activity of honey is based on multiple mechanisms and involves its osmotic activity, low pH, the formation of hydrogen peroxide, and the presence of various phytochemicals, such as alkaloids and flavonoids (caffeic acid, quercetin, pinobanksin, chrysin and galangin), and bee-added peptides, such as apidaecin and bee-defensin-1, that can exert an antimicrobial effect [14,15]. Antimicrobial resistance towards honey is not reported due to the versatility of the antimicrobial mechanisms [16]. The exact composition of honey highly depends on the geographic origin and the type of nectar, and as such, the antimicrobial activity can vary greatly between different types of honey [15]. L-Mesitran^®^ Soft, a formulation with 40% medical-grade honey, vitamins C and E, lanolin, and polyethylene glycol (PEG) 4000, is already in use as an ointment for wound care and in vitro studies suggest its activity against clinical *Candida* isolates [15]. The addition of vitamins and other components might be essential for the effectiveness of this honey [15,17,18]. Furthermore, the medical-grade honey in L-Mesitran^®^ Soft is carefully selected to minimize the presence of possible pollutants, while any dormant endospores that may be present are eradicated by gamma-sterilization [15]. The aim of this study is to investigate the susceptibility of *C. auris* and other *Candida* species to L-Mesitran^®^ Soft, whether antimicrobial activity can be attributed to the medical-grade honey component only and if another local unprocessed honey can exert similar effects.

## 2. Materials and Methods

### 2.1. Honey Samples and Preparation

CE- and FDA-approved L-Mesitran^®^ Soft [16,19] (formulation containing 40% medical-grade honey, hypoallergenic lanolin, propylene glycol, PEG 4000, and vitamins C and E), pure medical-grade honey following strict guidelines [10] (honey component of ^®^ L-Mesitran^®^ Soft, Brazilian blossom honey) and unprocessed (non-sterilized) local honey (provided by local beekeeper from Mook, the Netherlands) were included in this study. These compounds were suspended in 1× RPMI 1640 medium to reach final concentrations of 16% and 40% (*v*/*v*) after the addition of *Candida* suspensions. As the percentage of honey in L-Mesitran^®^ Soft is 40%, the final honey concentrations in the L-Mesitran^®^ Soft solutions of 16% and 40% (*v*/*v*) were 6.4% and 16%, respectively. Subsequently, a 16% solution of the two other honey products contains the same amount of honey as 40% L-Mesitran^®^ Soft.

### 2.2. Candida Strains

Clinical isolates from different geographic regions were selected for *C. auris*, *C. albicans*, *C. glabrata*, *C. krusei* and *C. parapsilosis* (Supplementary Table S1), while 32 *C. auris* isolates were used for tests on *C. auris* only. Isolates were stored at −80 °C according to standard procedures. Species identification via sequencing and/or matrix-assisted laser desorption ionization-time of flight mass spectrometry (MALDI-TOF MS) was conducted as described previously [20].

### 2.3. Testing Antifungal Effect Honey

RPMI 1640 medium (Gibco, Paisley, Scotland, UK) with different concentrations of the honey samples was added to Corning Costar cell culture cluster 96-well plates (Corning Costar Inc., Tewksbury, MA, USA) and stored at −80 °C until use. Strains were suspended in 2.5 mL sterile physiological saline and yeast quantity was determined using the Genesys 20 Spectrophotometer (ThermoSpectronic, UK). After dilution to 75–77% transmittance, corresponding to 1–5 × 10^5^ colony-forming units (CFU)/mL, suspensions were 100× diluted in RPMI 1640 media. These suspensions (100 µL) were added in triplicate to the Cornstar 96-well plate with honey suspensions (100 µL) and incubated in the O_2_ incubator at 35 °C for 24 h. Subsequently, these suspensions were streaked separately on sheep blood agar (ThermoFisher Scientific, Waltham, MA, USA) and incubated in the O_2_ incubator at 35 °C for 24 h.

### 2.4. Antifungal Susceptibility Testing

The in vitro antifungal susceptibility of 32 *C. auris* isolates (*n* = 32) was determined using the M38-A2 broth microdilution method of CLSI (Clinical and Laboratory Standards Institute) [5]. The antifungals tested included amphotericin B (Bristol Myers Squib, Woerden, The Netherlands), fluconazole (Pfizer Central Research, Sandwich, UK), itraconazole (Janssen Cilag, Beerse, Belgium), voriconazole (Pfizer Central Research), posaconazole (Merck, Sharp & Dome, Haarlem, The Netherlands), isavuconazole (Basilea Pharmaceutica, Basel, Switzerland), micafungin (Astellas Pharma, Toyama, Japan) and anidulafungin (Pfizer Central Research), all dissolved in DMSO. Isolates were resuspended in RPMI 1640 medium as described, transferred to microtitre plates with antifungals, incubated at 35 °C and analyzed visually after 24 h. Drug- and yeast-free controls were included. The MIC endpoints of all drugs except amphotericin B were defined as the lowest drug concentration that caused a prominent decrease in growth (50%) in relation to the controls and for amphotericin B, the MIC was defined as the lowest concentration at which there was 100% inhibition of growth compared with the drug-free control wells. 

### 2.5. C. auris DNA Isolation, Molecular Beacon-Based Melting Curve Analysis and Sequencing

Single colonies were resuspended in physiological saline with 200 U lyticase (Sigma-Aldrich, St. Louis, MO, USA) and incubated for 5 min at 37 °C, followed by 15 min at 100 °C. These DNA-containing solutions were used for the following molecular analyses. To analyze the presence of S639F in FKS1 in *C. auris*, a molecular beacon-based melting curve analysis was used with excess primer CauF1H1-X (5′-CGTCATGGTGGACAAGTTTCTA-3′), limiting primer CauF1H1-L (5′-GGGTCACTGTGTTTGCTGCTAAGTTGG-3′) and molecular beacon CauF1H1-WT (5′-FAM-CGCGACTTCTTGACTTTGTCCTTGAGAGATCCTGTCGCG-BHQ1-3′) [21]. The thermal program consisted of 3 min incubation at 95 °C; 45 cycles of 10 s at 95 °C, 20 s at 60 °C, and 30 s at 72 °C; a 2 minute incubation at 72 °C, and following amplification, samples were incubated for 2 min at 95 °C, and melted from 54 °C to 67 °C with a ramp rate of 0.025 °C/s [21], and was executed using a Thermocycler (Westburg, Biometra, Göttingen, Germany). PCR components included 1× Fast Start Taq polymerase buffer with MgCl2, 0.2 mM deoxynucleoside triphosphates (dNTPs), 1 U Faststart Taq polymerase (Roche Diagnostics, Germany), 1 µM CauF1H1-X, 40 nM CauF1H1-L, and 500 nM CauF1H1-WT, water, and DNA. For sequencing, the ERG11 region containing Y132 and K143 was first amplified using primers 5′-TGCTTATTCCCACTTGACCACTCCAG-3′ and 5′-ACTTCCTCTTGGATTCTGGG-3′ with the PCR components and program as described above with final concentrations of 0.5 µM for both primers. After amplification, sequencing was performed as described [22]. In short, after purification using the Ampliclean method (NimaGen, Nijmegen, The Netherlands), the sequencing PCR was performed using 0.5 μL BrilliantDye premix, 1.75 μL BrilliantDye 5× sequencing buffer (NimaGen), 5 pmol fwd or rev primer, 5.75 μL water, and 1 μL DNA. After D-Pure purification (NimaGen) sequencing was performed using the 3500XL genetic analyzer (Applied Biosystems, Foster City, CA, USA) and data were analyzed in Bionumerics 7.6.1 (Applied Maths, Kortrijk, Belgium).

### 2.6. Statistical Analysis

A 2-way ANOVA with Bonferroni’s multiple comparisons test was used to determine whether the effect of different honeys on *C. auris* were different from effects on other *Candida* species, while a 1-way ANOVA with Bonferroni’s multiple comparisons test was used for other analyses. Data was analyzed using Prism.

## 3. Results

### 3.1. Medical-Grade Honey Formulation Reduces C. auris Growth in a Concentration-Dependent Manner

**Figure 1:**
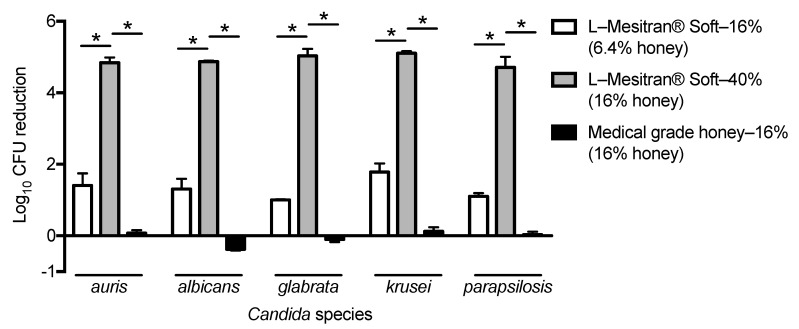
The effect of L-Mesitran^®^ Soft and its honey component on different *Candida* species. Isolates from five *Candida* species (*n* = 3 for each species) were incubated with L-Mesitran^®^ Soft—16% and —40% and medical-grade honey—16%. Data are presented as reduction in CFU. Significant differences (*p* < 0.05) are indicated with an asterisk.

To investigate the susceptibility of *C. auris* to the medical-grade honey formulation L-Mesitran^®^ Soft in comparison with other *Candida* species, we selected three isolates each of *C. auris*, *C. albicans*, *C. glabrata*, *C. krusei* and *C. parapsilosis* from different geographic regions. To test whether a potential susceptibility to the medical-grade honey formulation was largely due to the honey component or a synergistic effect of all ingredients together, the *Candida* isolates were also exposed to the pure medical-grade honey component only. After culture, resuspensions of these isolates were added to well plates with L-Mesitran^®^ Soft or medical-grade honey. A 24-h incubation with the medical-grade honey formulation induced a concentration-dependent reduction in *C. auris* CFU and the other *Candida* species (Figure 1). Surprisingly, a concentration of 16% medical-grade honey, which resembled the honey concentration in L-Mesitran^®^ Soft—40%, reduced the CFU of *C. auris*, *C. krusei* and *C. parapsilosis* less than 1-log, while it even stimulated growth in *C. albicans*, and *C. glabrata,* demonstrating that the honey component alone does not explain the effect of the medical-grade honey formulation (Figure 1).

### 3.2. Candida Growth Is Inhibited by Honey

**Figure 2:**
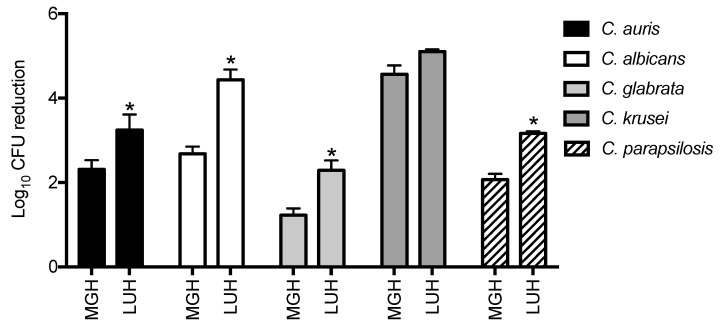
The effect of medical-grade honey vs local honey on the growth of different *Candida* species. Isolates from five *Candida* species (*n* = 3 for each species) were incubated with 40% of medical-grade honey (MGH) or local unprocessed honey (LUH). Data are presented as reduction in CFU. Significant differences (*p* < 0.05) as compared to MGH are indicated with an asterisk.

Next, we tested whether a 2.5-fold higher concentration of medical-grade honey (40%) affected the growth of *C. auris* and the other *Candida* species. The medical-grade honey in this concentration is also present in the undiluted medical-grade honey formulation. Furthermore, to determine whether the potential antifungal effect of the medical-grade honey was more effective than a random other honey, we compared its efficacy to unprocessed local honey. Medical-grade honey at a concentration of 40% led to a lower number of CFU for all *Candida* species, while this was also observed for the local unprocessed honey—40% (Figure 2). Comparing the effect of medical-grade honey with local unprocessed honey, we found that the latter honey was more effective in reducing all *Candida* species, except *C. krusei* for which no significant difference was found.

### 3.3. C. auris Susceptibility to Medical-Grade Honey Formulation Is Not Dependent on Genetic Origin but Seems Increased in Multiresistant Isolates

**Figure 3:**
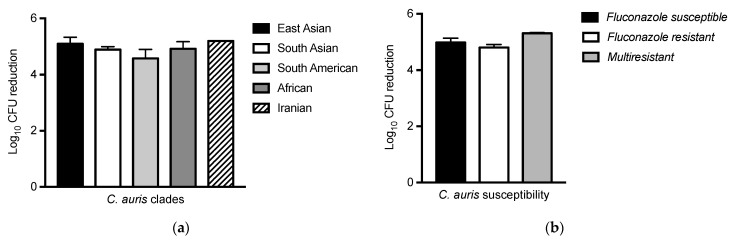
The impact of *C. auris* traits on antifungal activity of L-Mesitran^®^ Soft. *C. auris* isolates with (**a**) different geographic origins, and; (**b**) different antifungal susceptibilities, were incubated with L-Mesitran^®^ Soft—40%. Multiresistant isolates include those that are resistant to fluconazole and echinocandin and/or amphotericin B. No significant differences were found between groups.

To investigate whether the susceptibility of *C. auris* to the medical-grade honey formulation L-Mesitran^®^ Soft was dependent on its genetic origin or resistance to antifungals, we tested this medical-grade honey formulation at a concentration of 40% on 32 *C. auris* isolates originating from the five known *C. auris* clades and determined the antifungal susceptibility. No difference in susceptibility to the medical-grade honey formulation was found between the different clades (Figure 3a). Subsequently, analyzing the antifungal resistance, we found that out of the 32 isolates, 30 were resistant to fluconazole, 3 to amphotericin B, and 2 to echinocandins (Table 1). By investigating the molecular cause of fluconazole and echinocandin resistance via sequencing, we found that from the 30 fluconazole-resistant isolates, 13 harbored the Y132F mutation, and 8 the K143R mutation in *ERG11*. Using melting curve analysis, we found that the two isolates with echinocandin resistance both harbored the S639F mutation in *FKS1* (Supplementary Figure S1). Surprisingly, the melting curve of the Iranian isolate, in vitro susceptible to echinocandins, was also indicative of the S639F mutation in FKS1. Analyzing this region using whole-genome sequencing data [6] confirmed that this isolate indeed contains a SNP in the third nucleotide encoding S639, but this SNP did not alter amino acid coding and, as such, did not affect echinocandin susceptibility. No significant difference in growth was found in response to exposure with medical-grade honey formulation at a concentration of 40% when grouping the fluconazole susceptible isolates, those resistant to fluconazole, and the multiresistant isolates (resistant to fluconazole and echinocandin, or amphotericin B). However, the multiresistant isolates tended to be more susceptible to L-Mesitran^®^ Soft exposure (Figure 3b).

## 4. Discussion

In this study we found that the medical-grade honey formulation L-Mesitran^®^ Soft reduced *C. auris* CFU in a dose-dependent manner. Similar effects were found for *C. albicans*, *C. glabrata*, *C. krusei* and *C. parapsilosis*. L-Mesitran^®^ Soft consists of 40% medical-grade honey, hypoallergenic lanolin, propylene glycol, PEG 4000, and vitamins C and E. To understand whether the antifungal effect of this medical-grade honey formulation was mainly caused by the honey component, we exposed *C. auris* and the other *Candida* species also to the medical-grade honey component only. An identical concentration of honey as was present in the medical-grade honey formulation hardly reduced the growth of *C. auris*, *C. krusei* and *C. parapsilosis*, while it even stimulated the growth of *C. albicans* and *C. glabrata*. Thus, the honey component alone is not sufficient to mimic the effect of the medical-grade honey formulation on *C. auris*. The enhanced antimicrobial activity of L-Mesitran^®^ Soft has previously been attributed to the different supplements in the formulation [15,17,18,23]. Recently, a synergistic activity caused by the different ingredients of L-Mesitran^®^ Soft has been shown against *Pseudomonas aeruginosa* and *Staphylococcus aureus* biofilm formation and eradication [23].

To further analyze the effect of the honey component only, we investigated whether higher concentrations of honey would affect the growth of the different *Candida* species. Increasing the concentration of honey from 16% to 40% led to a 2-log reduction in the CFU of *C. auris*, while the CFUs of the other *Candida* species were also reduced by 1- to 4-log, with *C. glabrata* being least susceptible and *C. krusei* most susceptible. As previous studies found differences in the susceptibility of yeasts to different types of honey [11,12,13], the five *Candida* species were also treated with a local unprocessed honey. Local unprocessed honey (40%) caused a 2- to 5-log reduction in all *Candida* species. Thus, the local unprocessed honey was roughly 1-log more effective than the medical-grade honey in reducing *Candida* growth. This suggests that the antifungal constituents of the unprocessed local honey were more effective, or that the gamma-irradiation of honey reduced the efficacy of the medical-grade honey. A 100-fold difference in antimicrobial activity between honey types has been reported previously [24,25], but more interestingly, we here show that the antimicrobial activity of honey can be strongly enhanced by using supplements, such as those used in L-Mesitran^®^ Soft. Even at 6.25-fold dilution, this medical-grade honey formulation induced a more than 1-log reduction in all *Candida* strains. This medical-grade honey formulation effectively treats infected human wounds with (multiresistant) bacteria [16] and the canine otitis externa [26]. Therefore L-Mesitran^®^ Soft is a potential candidate for the treatment of wounds and skin colonized with *C. auris* and other *Candida* species.

Finally, we found that the susceptibility of *C. auris* to L-Mesitran^®^ Soft is not affected by its geographic origin, as the susceptibility of different clades to this medical-grade honey formulation did not differ. Resistance to fluconazole alone or combined with echinocandin and/or amphotericin B resistance did not affect the susceptibility of *C. auris* isolates to the medical-grade honey formulation, although the multiresistant isolates tended to be more sensitive. The increased susceptibility of multiresistant microorganisms to alternative treatments has been observed previously, and is known as collateral susceptibility [27,28,29]. Studies including larger numbers of multiresistant isolates should determine whether multiresistant *C. auris* also demonstrates collateral susceptibility.

## 5. Conclusions

*C. auris* and other *Candida* species are highly susceptible to medical-grade honey and local unprocessed honey in vitro. More interestingly, L-Mesitran^®^ Soft, a medical-grade honey-based wound care formulation, showed significantly higher antimicrobial activity, supporting the interpretation that the antimicrobial activity of medical-grade honey was strongly enhanced by different supplements. Therefore, this medical-grade honey formulation might therefore be a promising treatment for open wounds or skin colonized with *C. auris*. Future studies should demonstrate whether such treatment is feasible and effective in the clinical setting.

## Figures and Tables

**Table 1:** Overview susceptibility of clinical *C. auris* isolates with the corresponding mutations.

CWZ ID ^1^	Country	Clade	MIC for Different Antifungals (µg/mL) ^2^								Log Growth Reduction with L-Mesitran Soft ^3^	Sequencing/Melt Curve ^4^		
			AMB	FLC	ITC	VOR	POS	ISA	ANI	MICA		Y132	K143	S639
10-11-10-19	Netherlands	I	1	64	0.063	0.25	<0.016	<0.016	0.063	0.063	4.7	Y132F	WT	WT
10-11-10-29	Oman	I	2	16	<0.016	0.063	<0.016	<0.016	0.031	0.031	4.4	WT	K143R	WT
10-11-10-30	Oman	I	1	>64	0.25	1	0.063	0.125	0.063	0.063	5.2	WT	K143R	WT
10-11-13-25	India	I	1	64	0.125	0.125	0.031	0.031	0.063	0.063	4.6	WT	K143R	WT
10-04-18-46	India	I	1	>64	0.25	4	0.125	0.5	0.063	0.063	5.3	Y132F	WT	WT
10-12-18-19	India	I	1	>64	0.25	0.5	0.063	0.125	0.125	0.063	3.6	WT	WT	WT
10-12-12-01	Germany	I	1	16	<0.016	0.125	<0.016	<0.016	0.031	0.063	5.3	Y132F	WT	WT
10-12-12-02	Germany	I	1	8	0.063	0.125	<0.016	<0.016	0.125	0.125	4.2	Y132F	WT	WT
10-08-12-39	England	I	1	4	<0.016	0.031	<0.016	<0.016	0.031	0.031	5.1	Y132F	WT	WT
10-08-12-40	England	I	1	16	<0.016	0.125	<0.016	<0.016	0.125	0.063	4.8	Y132F	WT	WT
10-12-18-16	Pakistan	I	0.25	1	<0.016	<0.016	<0.016	<0.016	0.063	0.063	5.2	WT	WT	WT
10-12-18-17	Pakistan	I	1	>64	0.25	1	0.063	0.125	0.063	0.063	4.0	WT	K143R	WT
10-08-13-03	Kuwait	I	1	>64	0.25	1	0.063	0.25	0.125	0.063	5.2	WT	K143R	WT
10-08-13-46	Kuwait	I	1	>64	0.25	0.5	0.063	0.125	0.063	0.063	5.3	WT	K143R	WT
10-05-12-66	India	I	8	64	0.5	2	0.25	0.5	8	8	5.3	WT	K143R	S639F
10-05-12-72	India	I	2	64	0.125	16	4	4	0.125	0.125	5.3	Y132F	WT	WT
10-05-12-42	India	I	4	64	0.5	8	2	2	0.125	0.06	5.3	Y132F	WT	WT
10-05-12-62	India	I	0.5	64	0.125	2	0.03	0.25	8	8	5.3	WT	K143R	S639F
10-11-13-45	India	I	1	64	16	16	8	2	0.25	0.125	5.4	Y132F	WT	WT
10-03-10-62	South Korea	II	0.5	64	0.25	1	0.063	0.25	0.031	0.063	5.5	WT	WT	WT
10-03-10-63	South Korea	II	0.5	64	0.25	1	0.063	0.5	0.063	0.063	5.4	WT	WT	WT
10-12-18-10	Japan	II	0.5	1	<0.016	<0.016	<0.016	<0.016	0.063	0.063	5.1	WT	WT	WT
10-03-10-64	Japan	II	0.5	2	<0.016	<0.016	<0.016	<0.016	0.063	0.063	4.4	WT	WT	WT
10-05-15-49	South Africa	III	0.5	64	0.125	2	0.031	0.031	0.25	0.125	4.4	WT	WT	WT
10-05-15-50	South Africa	III	0.5	64	0.063	1	0.031	0.031	0.125	0.063	5.3	WT	WT	WT
10-08-12-09	Spain	III	0.25	>64	0.125	0.5	<0.016	<0.016	0.063	0.063	4.5	WT	WT	WT
10-08-08-21	Spain	III	0.5	64	0.125	1	0.063	0.063	0.063	0.063	5.4	WT	WT	WT
10-08-01-01	Venezuela	IV	0.5	>64	0.25	4	0.063	0.5	0.125	0.063	4.9	Y132F	WT	WT
10-08-01-02	Venezuela	IV	0.5	>64	0.25	4	0.125	0.5	0.125	0.125	4.1	Y132F	WT	WT
10-11-03-69	Colombia	IV	1	2	0.063	0.031	<0.016	0.031	0.125	0.063	5.3	WT	WT	WT
10-11-03-87	Colombia	IV	0.5	4	0.031	0.031	<0.016	0.031	0.063	0.063	4.0	WT	WT	WT
10-11-10-18	Iran	V	0.5	16	0.063	0.125	<0.016	0.063	0.016	0.031	5.2	WT	WT	WT *

^1^ CWZ ID refers to local identification number at CWZ (Canisius Wilhelmina Ziekenhuis). ^2^ CDC’s tentative breakpoints are ≥2 μg/mL for amphotericin B (AMB), ≥32 μg/mL for fluconazole (FLC), ≥4 μg/mL for echinocandins anidulafungin (ANI) and micafungin (MICA), while for the triazoles itraconazole (ITC), voriconazole (VOR), posaconazole (POS) and isavuconazole (ISA) no breakpoints are available. ^3^ Log growth reduction after treatment with L-Mesitran Soft 40%. ^4^ The presence or absence of Y132F/K143R and S639F mutations in ERG11 or FKS1, respectively. WT, wildtype. * Determined using WGS data [6] (melt curve analysis indicated the presence of S639F).

## Data Availability

The data presented in this study are available on reasonable request from the corresponding author.

## Supplementary Materials

The following are available online at https://www.mdpi.com/2309-608X/7/1/50/s1, Supplementary Figure S1. Normalized Melting Curves for *C. auris* isolates. Supplementary Table S1: Overview of strains for each *Candida* species.
