# Supplementary material for: Antifungal Activity of a Medical-Grade Honey Formulation against Candida auris
Source: J Fungi (Basel). 2021 Jan 13;7(1):50. doi: 10.3390/jof7010050 (PMC7828376; doi:10.3390/jof7010050)
Supplement: Supplementary file 1 [file jof-07-00050-s001.pdf]

**Supplementary data**

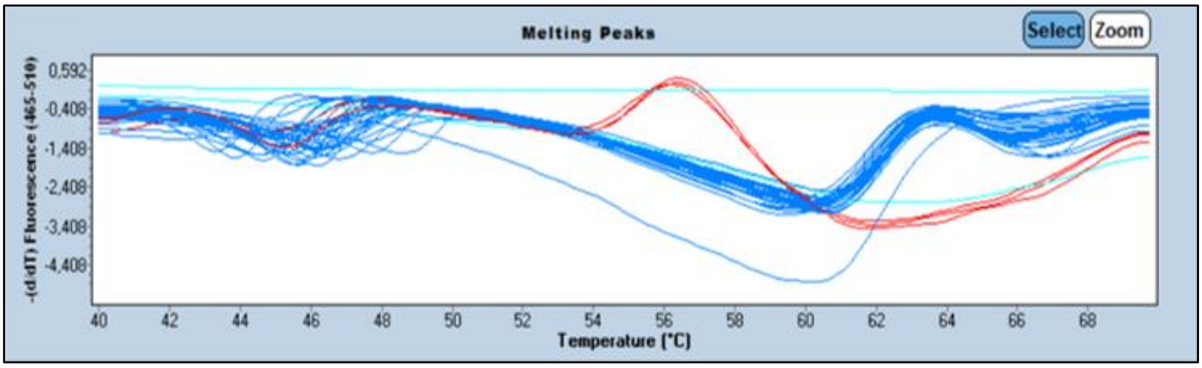


**Supplementary Figure S1. Normalized Melting Curves for *C. auris* isolates.** The FKS1 region containing S639 from 35 *C. auris* isolates was amplified via PCR followed by molecular beacon-based melting curve analysis using LightCycler® 480-II Instrument Software. Isolates with melting peaks around 56°C indicate the presence of S639F (red lines), while melting peaks around 61°C indicate the presence of WT FKS1 (blue lines).

**Supplementary Table S1. Overview of strains for each *Candida* species.**

| Strains | CWZ ID | Alternative number | Country of origin |
|---|---|---|---|
| *Candida auris* | 10-03-10-62 | KCTC 17809 | Korea |
| | 10-04-18-46 | VPCI 482/P/13 | India |
| | 10-08-01-02 | 10-08-01-02 | Venezuela |
| *Candida albicans* | 10-06-03-87 | 1082257609 | the Netherlands |
| | 08-44-07-01 | 154 | Scandinavië |
| | 10-03-10-06 | AM 07/0267 | England |
| *Candida glabrata* | 10-03-02-45 | 144 | Qatar |
| | 10-04-19-95 | IFRC 649 | Iran |
| | 10-07-03-99 | 5042020417 | the Netherlands |
| *Candida krusei* | 10-13-01-27 | VPCI-1623/P/16 | India |
| | 10-03-02-98 | 197 | Qatar |
| | 11-01-05-36 | ATCC 6258 | Sri Lanka |
| *Candida parapsilosis* | 10-07-15-18 | 8655 | Brasil |
| | 10-03-02-05 | 104 | Qatar |
| | 10-11-05-27 | VPCI 2289/P/16 | India |
